# AutoHMD: scalable and accurate validation of antibody–antigen complexes by heated molecular dynamics

**DOI:** 10.1093/bioinformatics/btag757

**Published:** 2026-10-09

**Authors:** Julia S Souza, Aline O Albuquerque, Diego S Almeida, Andrielly H S Costa, Jean V Sampaio, João H M Silva

**Affiliations:** Departamento de Bioquímica e Biologia Molecular, Universidade Federal do Ceará, Av. Humberto Monte s/n, Fortaleza, Ceará, 60020-181, Brazil; Structural and Functional Biology in Biopharmaceuticals Group, Fiocruz Ceará, R. São José, s/n, Eusébio, Ceará, 61773-270, Brazil; Structural and Functional Biology in Biopharmaceuticals Group, Fiocruz Ceará, R. São José, s/n, Eusébio, Ceará, 61773-270, Brazil; Structural and Functional Biology in Biopharmaceuticals Group, Fiocruz Ceará, R. São José, s/n, Eusébio, Ceará, 61773-270, Brazil; Postgraduate Program in Computational and Systems Biology, Oswaldo Cruz Foundation (Fiocruz), Av. Brasil, 4365, Rio de Janeiro, Rio de Janeiro, 21040-900, Brazil; Structural and Functional Biology in Biopharmaceuticals Group, Fiocruz Ceará, R. São José, s/n, Eusébio, Ceará, 61773-270, Brazil; Structural and Functional Biology in Biopharmaceuticals Group, Fiocruz Ceará, R. São José, s/n, Eusébio, Ceará, 61773-270, Brazil; Postgraduate Program in Computational and Systems Biology, Oswaldo Cruz Foundation (Fiocruz), Av. Brasil, 4365, Rio de Janeiro, Rio de Janeiro, 21040-900, Brazil; Structural and Functional Biology in Biopharmaceuticals Group, Fiocruz Ceará, R. São José, s/n, Eusébio, Ceará, 61773-270, Brazil; Pasteur–Fiocruz Center on Immunology and Immunotherapy, Oswaldo Cruz Foundation (Fiocruz), R. São José, S/N, Eusébio, Ceará, 61773-270, Brazil

## Abstract

**Motivation:**

Antibody–antigen (Ab–Ag) interactions are central to immune recognition and widely exploited in therapeutic design, making accurate structural characterization of these complexes a central challenge in the field. While docking and artificial intelligence–based approaches can generate multiple candidate binding modes, reliably identifying near–native conformations represent a key bottleneck. Existing methods depend largely on static scoring functions, which fail to capture the dynamic nature of Ab–Ag interfaces and, as a result, offer limited power to discriminate between competing poses.

**Results:**

Here, we evaluate heated molecular dynamics (HMD) as a strategy to discriminate binding pose quality based on structural stability under thermal perturbation, implemented through the automated workflow AutoHMD. Across a diverse set of Ab–Ag complexes, HMD enabled clear separation between high–quality and incorrect poses. During simulations, near–native complexes maintained a stable interface, whereas incorrect ones showed progressive divergence, particularly at elevated temperatures. Interaction analysis revealed that high-quality poses maintain more stable and persistent interfacial contacts throughout the simulations. In contrast, incorrect poses display an unstable interaction network. Additional simulation replicates increased classification consistency, while a mean iRMSD cutoff of approximately 2.5 Å achieved 90% accuracy in cross-validation and retained robust performance on an independent external set. Its transfer to Boltz-2-generated complexes further supported the broader applicability of HMD to AI-predicted binding poses. Overall, AutoHMD provides a reproducible, scalable, and high–throughput framework for validation of predicted antibody–antigen complexes, with potential applications in structural bioinformatics and biopharmaceutical design.

## Graphical abstract

**Graphical Abstract:**
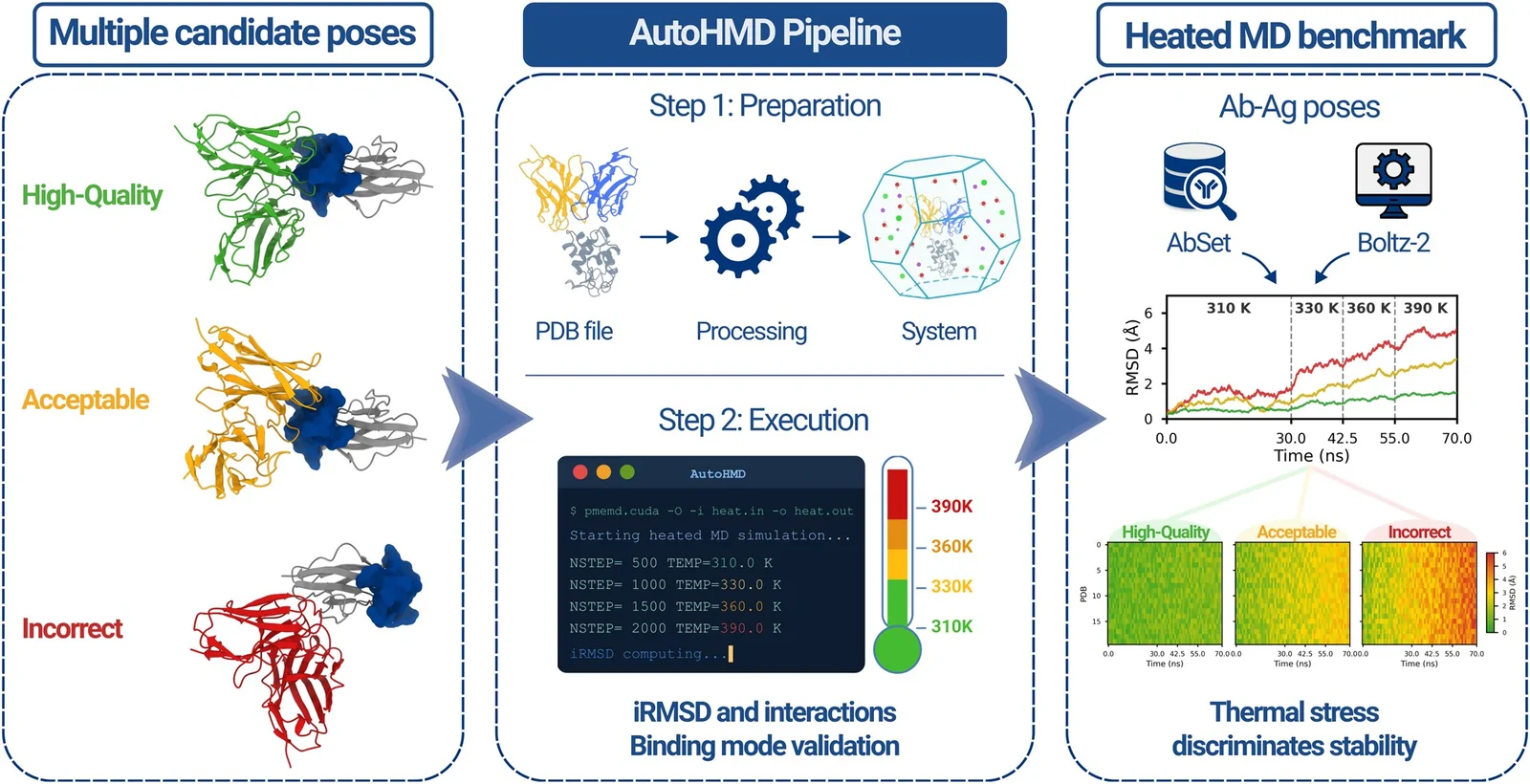

## 1 Introduction

Molecular interactions underpin most biological processes, from cellular signaling to immune recognition. The way biomolecules interact, associate, and dissociate governs the behavior and regulation of biological systems (Matamá *et al.* 2025). Among them, antibody–antigen (Ab–Ag) interactions are notable for their high affinity and specificity (Peng *et al.* 2014), largely driven by the complementarity-determining regions (CDRs), whose sequence and conformational diversity enable recognition of a wide range of antigens (Akbar *et al.* 2021, Mejias-Gomez *et al.* 2023).

Computational methods are increasingly used to reduce the time and cost of predicting Ab–Ag interactions (Sormanni *et al*. 2018). Among them, molecular docking approaches such as ZDOCK (Chen *et al*. 2003), HADDOCK (Dominguez *et al*. 2003), and ClusPro (Kozakov *et al.* 2017) combine conformational sampling with chemical, physical, or statistical scoring functions to evaluate generated binding poses. Although docking methods can generate near-native conformations, accurately ranking them among top-scoring solutions remains a major challenge (Janin *et al.* 2003, Guest *et al.* 2021). This difficulty is exacerbated by the intrinsic flexibility of antibody CDR loops, particularly H3, contributing to the limited correlation between energy- and artificial intelligence (AI)-based scoring functions and experimental binding energy and entropy data (Guest *et al.* 2021, Sampaio *et al.* 2026). More recently, deep learning methods such as AlphaFold3 (Abramson *et al.* 2024), Chai–2 (Team *et al.* 2025), and Boltz–2 (Passaro *et al.* 2025) have been developed for the prediction of protein–protein complex structures, with promising applications to Ab–Ag systems. However, their accuracy remains inconsistent, with AlphaFold3 exhibiting error rates of approximately 65% for Ab–Ag complexes, with similar limitations reported for Chai-2 and Boltz-2 (Hitawala and Gray 2025).

In this context, molecular dynamics (MD) simulations complement static docking approaches by explicitly accounting for molecular flexibility and enabling assessment of interfacial interaction persistence under near-physiological conditions. MD also enables binding free energy estimation through established end-point methods such as Molecular Mechanics/Generalized Born Surface Area (MM/GBSA) and Molecular Mechanics/Poisson–Boltzmann Surface Area (MM/PBSA), providing a thermodynamic basis for refining affinity predictions (Kollman *et al.* 2000, Genheden and Ryde 2015). However, these methods require sufficiently equilibrated trajectories to produce reliable free energy estimates, typically demanding extensive simulation times and substantial computational resources (Genheden and Ryde 2015).

Heated molecular dynamics (HMD) has emerged as a promising alternative for discriminating native from non-native docking poses within substantially shorter simulation timescales (Radom *et al*. 2018). The approach employs a controlled temperature increase during simulation to assess complex stability through interface RMSD (iRMSD). Although originally developed for general protein–protein complexes (Radom *et al*. 2018), HMD has also been successfully applied to Ab–Ag systems, including antibodies targeting integrin and CD3/CD117 (Chaves *et al.* 2020, Abdul Kadir *et al.* 2024), showing that non-native complexes destabilize as temperature increases. However, its generalizability remains unclear, as the method has been evaluated in only a limited number of cases.

This limited use likely reflects the complexity of MD simulations, which require extensive preparation, execution, and analysis involving multiple manual steps and custom scripts, reducing accessibility for researchers without programming expertise. To address this limitation, we developed AutoHMD, a workflow that automates the preparation, submission, and analysis of heated molecular dynamics simulations. AutoHMD was used to validate HMD across a diverse set of antibody–antigen complexes, including docking-generated structures and Boltz-2 models. HMD accurately distinguished high-quality from incorrect poses, with the former generally remaining below the iRMSD cutoff during heating and the latter exhibiting structural instability and exceeding the threshold. Based on these results, we propose an updated iRMSD cutoff for improved discrimination between near-native and incorrect poses. Together, these findings demonstrate the effectiveness of HMD as an evaluation strategy and the robustness of the AutoHMD workflow.

## 2 Methods

AutoHMD is an automated workflow that streamlines the preparation, execution, and analysis of HMD simulations for Ab–Ag complexes. The pipeline accepts a single Protein Data Bank (PDB) file containing the antibody heavy (H) and light (L) chains and the antigen, regardless of chain identifiers. AutoHMD automates structural preprocessing, protonation-state assignment, system parametrization and solvation, hydrogen mass repartitioning, molecular dynamics equilibration, heated production simulations, and post-processing analysis (Fig. 1). All stages are controlled through a user-editable configuration file (config_HMD.conf), enabling reproducible, scalable simulations with minimal user intervention. Version information for all third-party software is provided in the Supplementary Information.

**Figure 1:**
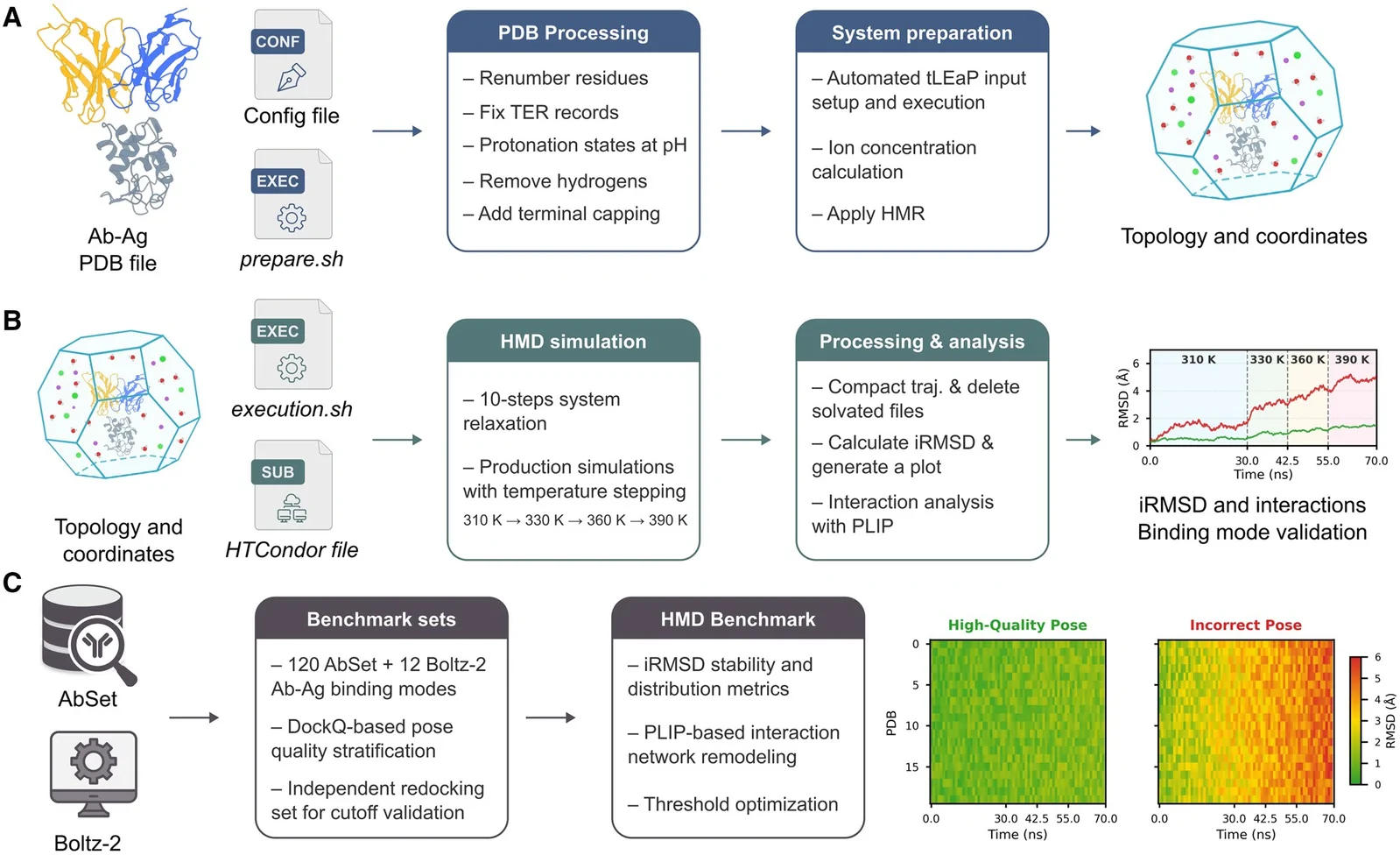
AutoHMD pipeline for Ab–Ag complex validation. (A) Automated system preparation starts from user-provided PDB and configuration files (Config file and prepare.sh), performing residue renumbering, pH-specific protonation, and solvation to generate MD-ready topology and coordinate files. (B) The execution script (execution.sh) automates relaxation and production MD with temperature stepping (310–390 K); an HTCondor file is provided for high-throughput submission. Automated iRMSD and optional PLIP analyses are then used to distinguish native binding modes from decoys. (C) Benchmarking strategy using 132 structures from AbSet and Boltz-2 classified as high-quality, medium, acceptable, or incorrect poses. Evaluation includes iRMSD analysis and descriptive statistics to characterize differences among pose-quality categories, followed by cross-validation to establish and evaluate classification cutoffs.

### 2.1 Input configuration

Simulation parameters are specified in the configuration file config_HMD.conf, located in the “prepare” directory. User-defined settings include the input PDB file, protein force field, water model, target pH for protonation-state assignment, solvation buffer distance, and ionic strength. System preparation is initiated by running prepare_autoHMD.sh. HMD simulations can be executed locally or through HTCondor-based high-throughput computing infrastructure. For local execution, a single script automatically imports the prepared topology and coordinate files. For cluster execution, a predefined HTCondor submission file is provided and can be adapted to local scheduling policies. Molecular dynamics input files are generated automatically, although simulation parameters may be customized before execution.

### 2.2 Structure preprocessing

Initial preprocessing of the input PDB file includes removal of malformed lines and insertion of TER records at sequence gaps to prevent artificial covalent bond formation during topology generation (Fig. 1A). Residues are then renumbered, and antibody insertion codes corrected using PDBTools (Rodrigues *et al.* 2018) to ensure consistent indexing throughout the workflow.

Protonation states are assigned at the user-specified pH, defaulting to 7.4 to approximate physiological conditions, using PDB2PQR (Dolinsky *et al.* 2007). Structures are then standardized for AMBER compatibility with PDB4AMBER and the Reduce utility from AmberTools (Case *et al.* 2023), ensuring consistent atom naming and hydrogen placement. To prevent artificial terminal charges, terminal capping groups are applied. For antibody Fv fragments, N-methylamine (NME) caps are added to the C-termini of heavy and light chains, reflecting their continuation in a full Fab context. Antigen chains receive acetyl (ACE) and NME caps at the N- and C-termini, respectively.

### 2.3 System building and parametrization

System assembly is performed with tLEaP from AmberTools. Proteins are parametrized using the force field specified in the configuration file, with ff19SB as the default (Tian *et al.* 2020). The complex is solvated in a truncated octahedral periodic box using a user-defined buffer, with the OPC water model used as the standard solvent representation. System charge is neutralized with Na^+^ and Cl^−^ counterions, followed by the addition of ions to achieve the target ionic strength, defaulting to 0.15 M. Hydrogen mass repartitioning (HMR) is applied with ParmEd, redistributing mass from heavy atoms to bonded hydrogens to enable a 4 fs integration timestep (Hopkins *et al.* 2015). Outputs include AMBER topology and coordinate files, a PDB file of the solvated system, and log files documenting the preparation steps (Fig. 1A).

### 2.4 Simulation protocol

All systems undergo a multi-stage relaxation and equilibration protocol before heated production simulations (Fig. 1B; see Supplementary Information for details). Production HMD is performed with the PMEMD engine of AMBER24 (Case *et al.* 2025). Temperature and pressure are controlled with the Langevin thermostat and Monte Carlo barostat, respectively. Following the protocol of Radom *et al.* simulations are conducted at 310 K for 30 ns, followed by sequential increases to 330 K and 360 K for 12.5 ns each, and 390 K for the final 15 ns (Radom *et al*. 2018). The default simulation length is 70 ns, although parameters can be modified by the user.

### 2.5 Trajectory processing and RMSD analysis

Trajectory processing and analysis are performed with CPPTRAJ (Fig. 1B). Individual simulation segments are concatenated into a single trajectory, and solvent molecules and ions are removed to reduce file size. iRMSD is calculated relative to the initial structure. Interface residues are defined as those with at least one atom within 8 Å of the binding partner in the reference structure. Outputs include processed trajectory and topology files, an iRMSD file, and a corresponding RMSD plot. Under default settings, the heated MD protocol generates 5600 trajectory frames; users modifying snapshot frequency or simulation length must update the plotting scripts accordingly. Additional scripts are provided for aggregation and visualization of iRMSD profiles from replicate simulations.

### 2.6 Interaction analysis

Additional interaction analysis can be performed using PLIP v2.3.1 (Schake *et al.* 2025) (Fig. 1B). All analysis parameters, including topology and trajectory files and sampling intervals, can be specified through either a configuration file or via the command line, with automatic identification and assignment of antibody and antigen chains. PLIP generates interaction maps and fingerprints describing hydrogen bonds, salt bridges, hydrophobic contacts, cation–π, and related non-covalent interactions. Residue numbering can be reassigned to user-defined standards, ensuring compatibility with external annotations.

### 2.7 Benchmark dataset and validation

The predictive power of the AutoHMD protocol was evaluated using a redocking ensemble derived from AbSet (Almeida *et al.* 2025), a curated dataset of standardized Ab–Ag complexes. To ensure structural diversity while maintaining a manageable benchmark, 30 PDB entries were selected (Fig. 1C; Table S1), spanning a broad range of targets. Antibody redundancy was assessed with MMSeqs2 Linclust (Mirdita *et al*. 2019). Only four anti-potassium channel KcsA and two anti-hen egg white lysozyme antibodies clustered as redundant, confirming a sequence-diverse antibody repertoire despite partial antigen recurrence. The inclusion of multiple complexes for some antigens may additionally capture variability in their structural recognition (Fig. S1).

Docking poses were classified into CAPRI categories using DockQ v2, which integrates iRMSD, ligand RMSD, and the fraction of native contacts relative to reference structures (Mirabello and Wallner 2024). For each complex, one pose from each CAPRI quality tier (high, medium, acceptable, and incorrect) was evaluated (Fig. S2). Boltz-2-generated models were also analysed to assess AutoHMD robustness on predicted Ab–Ag complexes. As no curated benchmark exists for AI-generated Ab–Ag models, systematic category-balanced assessments remain unavailable. Analysis of Boltz-2 models revealed a bimodal pattern, yielding either near-native poses closely matching crystal structures or no acceptable solutions. To address this limitation, we evaluated the discriminatory capacity of HMD on a heterogeneous dataset comprising 12 poses across 9 antigens (5 incorrect, 1 acceptable, and 6 high-quality) spanning multiple quality levels and antigen systems (Table S2).

Three independent replicates were performed for docking-derived poses and five for Boltz-generated models. Structural stability was assessed from the iRMSD profiles obtained throughout the HMD simulations. The mean, maximum, and standard deviation were calculated for each replicate and subsequently averaged for each pose. These iRMSD-derived descriptors were used for pose classification, with performance evaluated through leave-one-complex-out (LOCO) and leave-one-antigen-out (LOAO) cross-validation and further validated on an independent external set (Table S3). Classification accuracy and the Matthews correlation coefficient were used to assess predictive performance (see Supplementary Information for details).

The relationship between pose quality and different scoring approaches was additionally assessed. For docking-derived poses, DockQ was compared with the REF15 energy function implemented in PyRosetta and with scores from HADDOCK3, GNN-DOVE, and DeepRank-GNN-ESM (Table S4). While REF15 and HADDOCK Score provide an energy-based assessment (Alford *et al.* 2017, Giulini *et al.* 2025), GNN-DOVE and DeepRank-GNN-ESM employ graph-based representations, with the latter additionally incorporating protein language model embeddings (Wang *et al*. 2021, Xu and Bonvin 2024). For Boltz-2-generated models, AF3Score was used to obtain pDockQ, pDockQ2, pTM, and ipTM, complemented by ipSAE (Liu *et al.* 2025). HMD trajectories were assessed using the mean iRMSD over the complete trajectory and over the final 15 ns, corresponding to the 390 K stage. The association of each metric with DockQ was quantified using Spearman’s rank correlation.

To complement these stability descriptors, interfacial contacts were characterized with PLIP through the average number (*μ*) and duration of interactions, the number of unique residues involved, and persistent interactions, defined as those present in more than 50% of analysed frames. For this analysis, 175 frames per replicate were uniformly sampled from HMD trajectories. Structures were visualized using UCSF ChimeraX (Meng *et al.* 2023). To assess the contribution of selected interfacial residues to complex stability, alanine substitutions were introduced using UCSF Chimera 1.13, and the resulting mutants were resubmitted to the AutoHMD workflow.

## 3 Results

### 3.1 AutoHMD streamlines heated MD-based validation with minimal human intervention

The AutoHMD pipeline provides an optimized, fully customizable structure-preparation workflow driven by a single configuration file containing system-specific parameters, reducing manual intervention, minimizing errors, and improving reproducibility. Automation substantially increased scalability, enabling rapid preparation and submission of large simulation batches with minimal user input. Systems containing up to ∼900 residues were successfully processed, with runtimes ranging from ∼7 to 100 h depending on system size and GPU performance. Under these conditions, execution time was primarily limited by available computational resources, particularly GPU capacity (Fig. S3). Overall, the protocol achieved high throughput, supporting the execution of almost 400 independent HMD simulations of docked or modeled Ab–Ag complexes.

### 3.2 Heated molecular dynamics discriminates docking pose quality through interface stability

The HMD protocol effectively discriminated structurally correct from incorrect docking poses in a temperature-dependent manner, with high- and medium-quality poses remaining stable throughout heating (Fig. 2A–D). This behavior is consistent with their relatively low initial deviation from the crystallographic reference (mean iRMSD of 0.84 and 1.40 Å, respectively), compared with 3.29 Å for acceptable and 12.43 Å for incorrect poses (Fig. S4).

**Figure 2:**
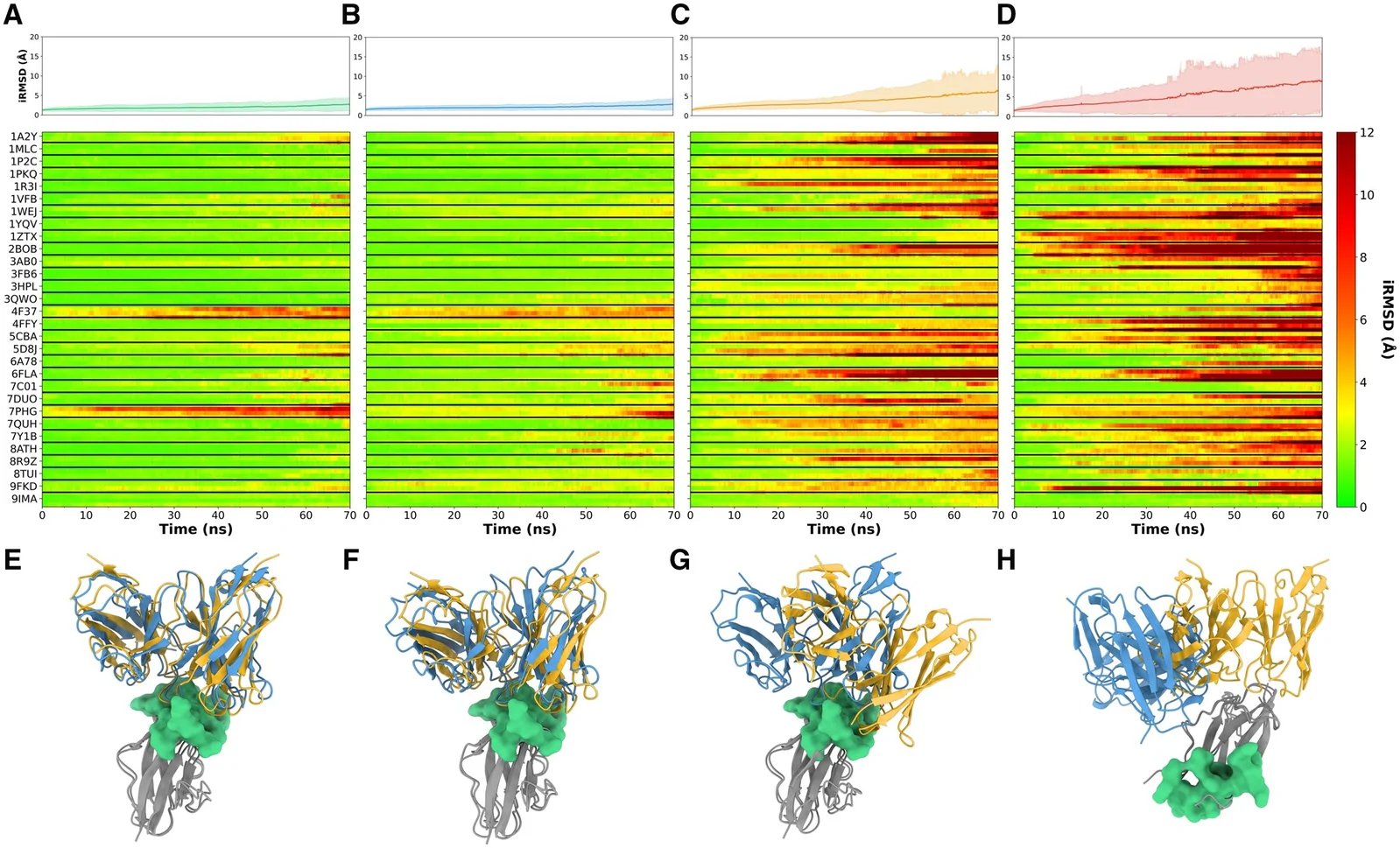
Stability benchmarking and structural validation of docked Ab–Ag poses. (A–C) Heatmaps showing iRMSD profiles over 70 ns of HMD simulations for (A) high-quality, (B) medium (C) acceptable, and (D) incorrect poses. The green-to-red gradient indicates increasing interfacial deviation and binding mode instability. (E–H) Structural snapshots of the West Nile Virus E protein–E16 antibody complex (PDB ID: 1ZTX), comparing the initial docked structure (blue) and final HMD conformation (yellow) relative to the native epitope (green surface). Panels show (E) high- and (F) medium-quality pose preservation, (G) acceptable pose deviation, and (H) incorrect pose displacement.

In contrast, incorrect poses exhibited marked interface instability, with most trajectories exceeding the 5 Å cutoff proposed by Chaves *et al.* (2020) early in the simulations and remaining above it thereafter. Acceptable-quality poses displayed intermediate behavior. Although they remained relatively stable at lower temperatures, most trajectories destabilized after the temperature increase to 360 K and became more clearly separated from high- and medium-quality poses during the final 390 K stage.

Overall, thermal stress enhanced the distinction between correct and incorrect poses (Fig. S5). Since acceptable-, medium-, and high-quality poses all recover the correct binding mode, differing primarily in interface accuracy, the AutoHMD workflow provides a complementary filtering step focused on separating structurally plausible binding modes from incorrect predictions. Consistent with this discriminatory behavior, HMD-derived iRMSD showed the strongest association with DockQ among the evaluated methods, with Spearman correlations of −0.73 for the complete trajectory and −0.75 for the final 15 ns at 390 K, compared with −0.62 for HADDOCK score, −0.04 for REF15, 0.20 for DeepRank-GNN-ESM, and −0.18 for GNN-DOVE (Figure S6). This discriminatory capacity is exemplified by the poses from PDB ID: 4FFY, for which REF15, DeepRank-GNN-ESM, and GNN-DOVE all favored the incorrect binding mode over the native one. In contrast, HMD clearly separated the two, with the native pose remaining close to the reference (mean iRMSD 1.5 Å) while the incorrect pose drifted substantially (10.5 Å), illustrating how dynamic stability can distinguish poses not resolved by static scoring functions.

Two high-quality systems deviated from the overall stability trend. Poses derived from PDB IDs 4F37 and 7PHG exceeded the 5 Å cutoff early and displayed pronounced iRMSD fluctuations. HMD simulations of the corresponding crystal structures reproduced this behavior, with iRMSD values exceeding 8 Å (Fig. S7), indicating that the instability was intrinsic to the complexes rather than a simulation artifact. Notably, the P5C3 antibody (PDB ID 7PHG) is a high-affinity SARS-CoV-2 RBD binder, with EC80 values of 0.02–0.035 μg/mL (Fenwick *et al.* 2021), suggesting that thermal stability and binding affinity, although often correlated, are not necessarily coupled. Because HMD probes the kinetic robustness of a binding pose under thermal stress rather than equilibrium binding thermodynamics, even experimentally validated high-affinity complexes may appear unstable. For computational poses, this behavior may also result from inaccuracies in interface geometry or binding mechanisms that depend more strongly on entropic and water-mediated contributions. In the anti-amyloid beta antibody (PDB ID 4F37), which binds a short N-terminal antigen segment of approximately 10 residues, the limited interface likely contributes to the elevated flexibility observed during HMD.

A different behavior emerged for the CB6–RBD (PDB ID 7C01) and talquetamab–GPRC5D systems (PDB ID 9IMA), which remained stable despite thermal stress. In the CB6–RBD complex, the high-quality pose displayed an interaction network centered on CDR-H3 Asp100A and CDR-L3 Tyr92, which formed a salt bridge with Lys417 and a cation–π interaction with Arg403, respectively (Fig. S8). Both antigen residues are key interaction hotspots in the crystallographic CB6–RBD interface (Shi *et al.* 2020). Although surrounding contacts varied across binding modes, both residues remained engaged at the interface: Tyr92 interacted with Arg403 in the acceptable pose and through hydrophobic contacts in the incorrect pose, whereas Asp100A formed a hydrogen bond with Asn440. Alanine substitution of these hotspots did not disrupt the high-quality complex, whereas the incorrect pose progressively destabilized during heating (Fig. S8). This resilience suggests that the native-like interface is supported by a redundant interaction network capable of compensating for individual contact disruption, including backbone-mediated hydrogen bonds retained after the CDR-H3 substitution.

The talquetamab–GPRC5D complex (PDB ID 9IMA) displayed a distinct basis for thermal stability. Its interface contained an extensive interaction network centered on Arg98, involving salt bridges, hydrogen bonds, and hydrophobic contacts with Asp238, Asp239, Asn167, Val159, Tyr93, Pro163, Leu166, and Phe158 (Fig. S8). Most interactions were preserved in the medium-quality pose, whereas the acceptable and incorrect ones retained the interaction with Asp239 but reorganized the remaining contacts. Arg98-to-alanine substitution did not destabilize either binding mode, indicating that stability was not dependent on this hotspot alone. Instead, resistance to thermal perturbation may reflect the comparatively rigid, predominantly α-helical architecture of the GPRC5D interface, highlighting a potential limitation of HMD discrimination for structurally constrained interfaces.

Despite these system-specific exceptions, descriptive analysis of the iRMSD trajectories supported the overall separation between structurally stable and unstable poses observed in the heatmaps (Fig. S9A–C). High-quality poses consistently showed lower mean and maximum iRMSD values (1.93 and 3.72 Å, respectively) and a lower standard deviation (0.46 Å), reflecting structural stability and limited fluctuation during heating. In contrast, incorrect poses exhibited higher and more dispersed iRMSD values, with a mean of 5.10 Å, maximum of 12.77 Å, and standard deviation of 2.69 Å, consistent with progressive structural disruption. Redocked poses of the West Nile Virus E protein–E16 antibody complex (PDB ID: 1ZTX) (Fig. 2E–H) exemplified these trends. The high-quality pose showed minimal deviation between the initial docking structure (blue) and final MD conformation (yellow), preserving the native binding mode over the epitope (green surface). The acceptable pose displayed noticeable reorientation, whereas the incorrect pose exhibited extensive displacement and loss of the original interface, with the final structure markedly diverging from the initial configuration.

Moreover, the number of replicates directly influenced classification robustness (Fig. S9D). Although a single HMD replicate already provided substantial discriminatory information, achieving 81.7% accuracy in distinguishing incorrect from high-quality poses using a 5 Å maximum iRMSD cutoff, increasing the number of replicates to three raised the accuracy to 86.7%. This modest improvement suggests that a single trajectory may be sufficient for initial triage, while replicate simulations can reduce the influence of trajectory-specific fluctuations.

### 3.3 Thermal stress exposes persistent and transient interface signatures in docking poses

Interaction analysis along the trajectories revealed distinct interfacial patterns among pose categories. Incorrect and acceptable poses formed more diffuse and less specific contact networks, increasing the number of transient contacts sampled during simulations. This higher interaction count (acceptable *μ* = 183.0, incorrect *μ* = 195.6; Fig. S10A) likely reflects displacement of the Ab–Ag interface, promoting additional contacts as the system compensates for newly exposed interfacial residues. In contrast, high- and medium-quality poses exhibited significantly fewer interactions (*μ* = 118.4 and *μ* = 123.6, respectively; *P* < .001).

A similar trend was observed for the number of unique interacting residues (high-quality *μ* = 30.4; medium quality *μ* = 30.9; acceptable *μ* = 37.0; incorrect *μ* = 39.4; *P* < .01), whereas the opposite pattern was observed for mean interaction duration (Fig. S10B and C). High-quality complexes showed substantially longer interaction lifetimes, averaging 15.5% of analysed frames, compared with acceptable (6.2%) and incorrect poses (4.1%) (*P* < .001). Acceptable complexes also exceeded incorrect ones (*P* < .05). Consistently, analysis of persistent interactions (Fig. S10D), defined as those present for ≥50% of the simulation, showed that high-quality poses (*μ* = 11.5) maintained significantly more persistent contacts than acceptable (*μ* = 3.3) or incorrect ones (*μ* = 1.5) (*P* < .001). Together, these results indicate that although high-quality poses exhibit fewer total interactions and unique interacting residues, they form more stable and persistent interfacial contacts. Conversely, acceptable and incorrect poses contacts are transient and progressively disrupted during heating.

This behavior was consistent across systems, although the distinction between pose-quality categories emerged at different stages of the simulations (Fig. S11). In some cases (e.g. PDB IDs 8ATH and 3HPL), high-quality and acceptable poses were already markedly distinct, whereas in others (e.g. PDB ID 7C01), interaction loss in acceptable poses occurred only at higher temperatures despite a similar overall number of contacts to high-quality structures (25.2 vs 24.6), highlighting the role of thermal stress. In contrast, the incorrect pose of 7C01 exhibited fewer interactions from the outset. This trend extended to other incorrect poses, which not only maintained fewer interactions per frame but also showed greater variability in their interaction profiles, as observed for PDB ID 8R9Z (Fig. S11).

### 3.4 Optimization of iRMSD–based thresholds maximizes pose classification performance

A 5 Å iRMSD cutoff, previously adopted for antibody–integrin complexes (Chaves *et al.* 2020), was initially used as the reference threshold for pose classification. However, reported thresholds vary across systems; e.g. a 7 Å cutoff has been applied in anti-CXCR2 antibody studies (Che Nordin *et al*. 2026), highlighting the absence of a standardized criterion. Notably, these approaches rely exclusively on maximum iRMSD values and do not systematically evaluate alternative descriptors. To address this limitation, we assessed mean, maximum, and standard-deviation iRMSD as pose-classification descriptors across the CAPRI quality boundaries, selecting each threshold on a subset of complexes and evaluating it by leave-one-complex-out (LOCO) and leave-one-antigen-out (LOAO) cross-validation to avoid circular threshold selection.

For the primary distinction between high-quality and incorrect poses, mean iRMSD achieved 90.0% accuracy (MCC = 0.80) under both LOCO (95% CI, 0.667–0.935) and LOAO (95% CI, 0.592–0.946) cross-validation, with a stable threshold of 2.52 ± 0.02 Å (Table S5). The identical performance under both partitioning strategies indicates that discrimination was not dependent on antigen redundancy and was retained for antigens excluded from threshold optimization. This generalizability was further supported by the external redocking set, where the same threshold yielded 95.2% accuracy (MCC = 0.91), including 94.7% accuracy (MCC = 0.90) when restricted to novel antigens. Maximum iRMSD showed lower performance at the cross-validated operating threshold but maintained high discrimination between approximately 5.6 and 6.2 Å (Fig. 3). This lower sensitivity to cutoff selection is also reflected by the previously adopted 5 Å threshold, which, despite falling below the optimum identified here, still achieved 86.7% accuracy (MCC = 0.74). Thus, mean iRMSD provides a more defined threshold for binary classification, whereas maximum iRMSD remains informative across a broader interval.

**Figure 3:**
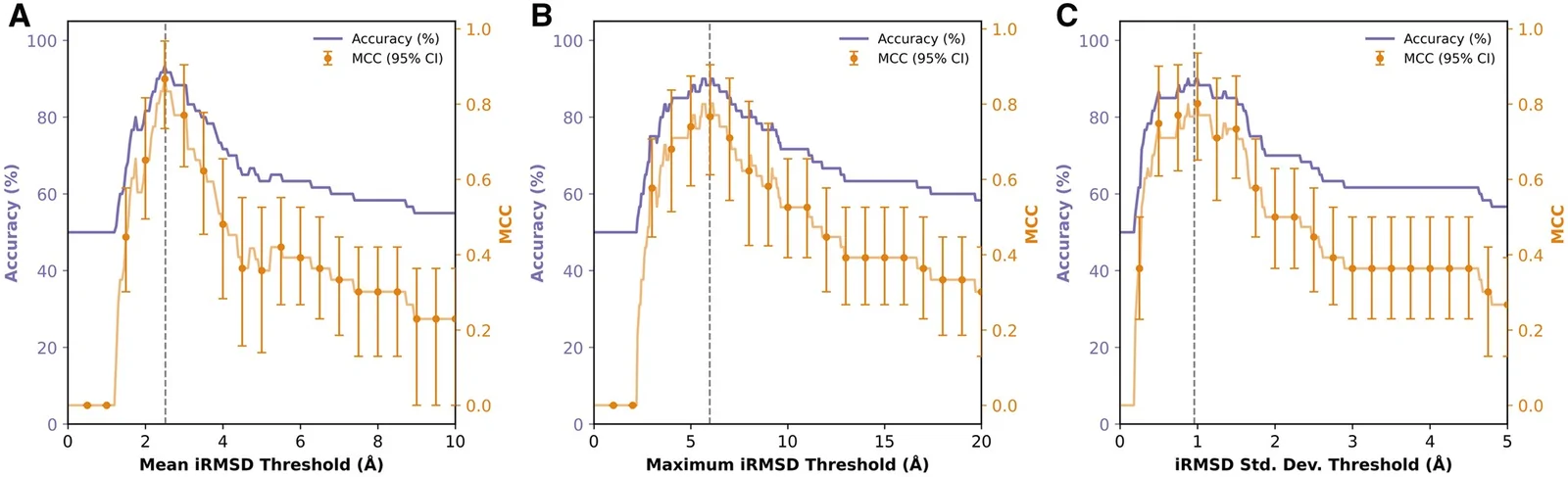
Classification performance across iRMSD thresholds. Accuracy (purple) and MCC (orange, mean ± 95% confidence interval from bootstrap over complexes) as a function of the (A) mean, (B) maximum, and (C) standard-deviation iRMSD threshold, for the high-quality versus incorrect comparison. Vertical dashed lines mark the cross-validated cutoffs.

When distinguishing between individual CAPRI categories, the descriptors provided complementary information. Maximum and mean iRMSD separated high-quality from acceptable poses with MCC values of 0.64 and 0.57, respectively (Table S5). At the acceptable-versus-incorrect boundary, which defines the CAPRI criterion for a successful prediction, mean iRMSD showed poor discriminatory power and yielded an unstable threshold. In contrast, iRMSD standard deviation achieved 73.3% accuracy (MCC = 0.48) at a threshold of 1.14 Å, which remained unchanged across folds and was retained on the external set. This shift in descriptor performance indicates that trajectory variability, rather than mean interface displacement, becomes more informative for distinguishing acceptable from incorrect poses. Discrimination between high- and medium-quality poses remained limited, consistent with their structural similarity.

Threshold-independent receiver operating characteristic (ROC) analysis further supported the complementary value of the iRMSD descriptors (Fig. S12). Mean and maximum iRMSD showed similar discrimination between high-quality and incorrect poses (AUC = 0.933 and 0.948, respectively), with overlapping confidence intervals. Mean iRMSD also yielded a slightly higher AUC for high-quality versus acceptable poses (0.904 versus 0.878), whereas maximum iRMSD yielded a higher AUC at the acceptable-versus-incorrect boundary (0.716 versus 0.669). Standard-deviation iRMSD showed a similar advantage at this boundary (AUC = 0.729), indicating that descriptors capturing larger structural excursions or trajectory variability provide additional information near the CAPRI success threshold. In contrast, discrimination between high- and medium-quality poses remained limited for all descriptors (AUC ≤ 0.68). Overall, mean iRMSD provides a more defined operating threshold for binary classification, with performance maintained across antigen-based partitioning and independent novel antigens, whereas maximum iRMSD offers comparable overall ranking ability and greater tolerance to cutoff selection. Together, these properties support the use of HMD-derived descriptors for pose classification beyond the systems used for threshold optimization.

### 3.5 AutoHMD differentiates AI-modeled binding poses generated by boltz–2

AI-based approaches are rapidly advancing protein–protein complex prediction. However, the unique features of antibody–antigen interfaces remain challenging, particularly for distinguishing accurate from incorrect models. HMD trajectories showed a consistent trend across the Boltz-2-generated models (Fig. S13). This distinction was further reinforced by descriptive statistics, as high-quality and acceptable poses displayed comparable mean iRMSD values (1.47 and 1.56 Å, respectively), contrasting with the substantially higher values observed for incorrect poses (3.83 Å) (Fig. S14A–C). These differences became more pronounced as temperature increased (Fig. S14D), indicating that thermal perturbation also exposes differences in the stability of AI-generated binding modes.

To determine whether this behavior could be captured without deriving model-specific criteria, the cutoffs established on the docking benchmark were evaluated directly on the Boltz-2 models. Due to the limited number of models, the benchmark-derived cutoffs were kept fixed rather than reoptimized on the Boltz-2 set. Mean iRMSD showed the highest transferability, with the 2.52 Å cutoff providing greater discrimination than maximum and standard deviation iRMSD (Fig. S15). High-quality models were concentrated at lower mean iRMSD values, whereas most incorrect models occurred above the cutoff, with some overlap between the two groups. This overlap was more pronounced for maximum and standard-deviation iRMSD, resulting in lower classification performance with the benchmark-derived cutoffs.

The relative performance of the descriptors was maintained across different numbers of HMD replicates (Fig. S15). Mean iRMSD consistently provided the highest accuracy and MCC, while increasing the number of trajectories produced only modest changes in classification performance, reducing variability across replicate combinations. Performance largely stabilized by three simulations, with only minor changes observed with the fourth and fifth replicates. Together, these results indicate that mean iRMSD transfers more consistently from redocked to Boltz-2-generated complexes, whereas maximum and standard-deviation iRMSD are more dependent on the dynamic behavior of individual models and do not recover comparable discrimination through additional sampling. Nevertheless, a comprehensive and category-balanced benchmark of AI-generated antibody–antigen complexes will be required to support robust threshold optimization for predicted structures.

Interaction analyses of Boltz-derived complexes revealed patterns consistent with those observed for docking poses. High-quality models exhibited fewer total interactions (*μ* = 126.5) than acceptable (*μ* = 137.0) and incorrect poses (*μ* = 205.2) (Fig. S16A), as well as fewer unique interacting residues (*μ* = 31.5, 29.0, and 38.2, respectively; Fig. S16B). In contrast, interaction duration (Fig. S16C) was higher in high-quality complexes (*μ* = 15.33%) than in incorrect (*μ* = 4.83%) and acceptable poses (*μ* = 9.01%). Likewise, persistent interactions (Fig. S16D) were enriched in high-quality poses (*μ* = 12.33) relative to incorrect (*μ* = 2.6) and acceptable complexes (*μ* = 3.0), representing an approximate fivefold increase (*P* < .01).

Although these observations should be interpreted cautiously given the limited sample size, which may restrict generalizability, these results recapitulate the trends observed for docking-derived poses: high-quality complexes, despite forming fewer interactions, maintain more stable and persistent interaction networks under thermal stress. In contrast, incorrect poses exhibit larger but transient networks that rapidly deteriorate during simulation, reflecting reduced structural stability.

## 4 Discussion

In this study, we demonstrated that heated molecular dynamics provides a robust strategy for discriminating antibody–antigen poses based on their stability under thermal perturbation. Large-scale application was enabled by AutoHMD, which automates system preparation, simulation, and analysis within a standardized framework, reducing user intervention, improving reproducibility, and supporting high-throughput applications. Using this protocol, 360 trajectories were generated and analysed for docking-derived poses, showing that correct binding modes maintained structural stability and persistent interfacial interactions even at elevated temperatures. This supports the notion that near-native complexes occupy energetically favorable conformations that remain stable under perturbation. In contrast, incorrect and acceptable poses exhibited pronounced interface instability, with more numerous but generally weaker, less specific, and progressively disrupted interactions, ultimately leading to destabilization.

The progressive disruption of interactions demonstrates the ability of HMD to distinguish biologically relevant binding modes and reflects how thermal perturbation amplifies subtle structural differences by probing kinetic stability. Near-native complexes, typically associated with deeper free-energy minima and more cooperative interaction networks, are more resistant to disruption, whereas incorrect poses often occupy metastable states and dissociate more readily despite their greater number of interactions. Such cooperativity was observed in the CB6–RBD complex (PDB ID 7C01), where the correct binding mode compensated for the simultaneous disruption of two interfacial hotspots through alternative contacts, while the incorrect pose destabilized following the same mutations. This differential response to perturbation is consistent with studies linking resistance to perturbation to lower dissociation rates (k_off_) in temperature- and force-based MD approaches (Pavan *et al.* 2022, Wang *et al*. 2024). Increasing temperature can therefore challenge interface integrity, as reported for protein–protein and antibody–integrin complexes (Radom *et al*. 2018, Chaves *et al.* 2020).

The strongest discrimination capacity was observed between 360 and 390 K in antibody–antigen systems. However, our results suggest that structural stability and binding affinity, although often correlated, are not always directly aligned. For example, PDB ID 7PHG exhibited reduced structural stability despite its high experimental affinity, whereas PDB ID 4F37 showed increased flexibility, likely due to its smaller interface. These observations suggest that affinity and structural stability may be influenced by distinct factors. Although mechanisms such as binding-induced folding (Sugase *et al*. 2007) or entropic penalties (Tzeng and Kalodimos 2012) could contribute to this decoupling, their involvement remains to be established. Despite the limited number of cases, these findings indicate that high-affinity interactions may involve flexible or transient binding modes, a consideration particularly relevant to antibody design.

Time-resolved iRMSD analysis proved a powerful discriminator of binding pose quality, consistent with previous MD-based studies. Jandova *et al.* (2021) showed that near-native protein–protein complexes display greater structural stability during 100-ns simulations, retaining a substantial fraction of native contacts, whereas non-native models lose most interactions over time, clearly separating correct and incorrect binding modes (Jandova *et al*. 2021). Extending these observations to antibody–antigen systems, our results show that HMD-derived descriptors not only distinguish high-quality from incorrect poses but also partially discriminate acceptable poses by capturing intermediate stability profiles. These findings reinforce the value of time-resolved metrics, including iRMSD trajectories, interaction duration, and persistence, over static scoring approaches that fail to capture system dynamics. This is consistent with recent work highlighting the importance of dynamic features for pose discrimination (Richard and Pantazes 2025) and with the superior performance of HMD-derived descriptors over the energy- and AI-based scoring methods evaluated here. Given the trajectory-dependent nature of these descriptors, replicate simulations provide an additional consideration, as different initial velocities allow sampling of distinct regions of the free-energy landscape (Knapp *et al*. 2018). In our analysis, additional replicates primarily reduced variability in classification across trajectory combinations, which was pronounced with a single trajectory but largely stabilized by three replicates. Mean classification accuracy, however, changed only modestly with additional sampling.

The use of complementary metrics, including MCC and ROC analysis, enabled a comprehensive assessment of structural deviation and systematic optimization of iRMSD thresholds. Within this framework, the three iRMSD descriptors provided complementary information. Mean iRMSD showed the strongest and most transferable discrimination between high-quality and incorrect poses, whereas standard deviation was more informative at the acceptable-versus-incorrect boundary. We further showed that HMD extends beyond docking-derived poses to AI-based models (Boltz-2), particularly through mean iRMSD assessment. However, these results should be interpreted cautiously, as AI-generated models may produce interaction patterns distinct from physically derived docking poses, potentially yielding stable yet non-native interfaces. This may reflect biases introduced during training on large structural datasets, leading to biologically incorrect but energetically favorable interfaces. Additionally, co-folding during prediction may further stabilize such complexes by simultaneously optimizing subunit conformations and the interface (Evans *et al.* 2021, Bryant *et al*. 2022). Consequently, even incorrect poses may maintain relatively stable interaction networks during heating, unlike docking-derived poses, which depend more directly on energetic and structural complementarity.

Some limitations should be considered. First, HMD simulations remain computationally demanding and dependent on GPU resources. Although AutoHMD streamlines system preparation and analysis, runtime is still constrained by hardware availability and the need for replicate simulations. Second, despite its relative breadth, the dataset may not fully capture the diversity of antibody–antigen interfaces, as illustrated by cases such as 7PHG and 4F37. This limitation is even more pronounced for Boltz-2 models, where the lack of standardized antibody–antigen benchmarks hampers the selection of balanced test sets. Consequently, dataset size and composition may introduce bias and limit the generalizability of the results.

## 5 Conclusion

In summary, we present an effective strategy for antibody–antigen binding mode validation that addresses key limitations of conventional scoring approaches. By leveraging thermal perturbation, HMD provides a reproducible and efficient framework for pose discrimination, capturing differences in interface stability and dynamics not readily apparent from static descriptors. Importantly, the temperature-ramping protocol offers a practical advantage over enhanced-sampling techniques by avoiding the need to define collective variables, a non-trivial and system-dependent task. Instead, structural integrity is monitored through simple geometric descriptors such as interface RMSD, facilitating implementation and reproducible analyses.

The scalability of this approach is supported by AutoHMD, which standardizes system preparation, simulation, and analysis for high-throughput applications. Although the current implementation relies on AMBER-based molecular dynamics, the workflow is modular and can be adapted to other engines such as GROMACS and NAMD. Future developments may further improve discrimination by integrating machine learning approaches, including random forest classifiers, to combine multiple descriptors and enhance pose ranking. Together, these features position HMD as a versatile and accessible tool for accelerating structural validation workflows and improving the reliability of computational predictions in applications such as biopharmaceutical design. In this context, AutoHMD serves as a valuable post-processing and quality-control step, helping identify false-positive poses generated by conventional scoring methods and neural network-based predictors that appear energetically favorable but remain kinetically unstable.

## Supplementary Material

btag757_Supplementary_Data

## Data Availability

The workflow script are available in GitHub at https://github.com/SFBBGroup/AutoHMD. The version of the code used in this study is permanently archived in Zenodo at https://doi.org/10.5281/zenodo.22757672. All simulation and structural data supporting this study are publicly available in Zenodo at https://doi.org/10.5281/zenodo.20722229; https://doi.org/10.5281/zenodo.22750139; https://doi.org/10.5281/zenodo.20707612.
