# Supplementary material for: AutoHMD: scalable and accurate validation of antibody–antigen complexes by heated molecular dynamics
Source: Bioinformatics. 2026 Oct 9;42(10):btag757. doi: 10.1093/bioinformatics/btag757 (PMC13693361; doi:10.1093/bioinformatics/btag757)
Supplement: Supplementary file 1 [file btag757_supplementary_data.docx]

**Supplementary Information**

**Software and dependencies**

AutoHMD was implemented and tested using Python 3.12. External dependencies include AmberTools and AMBER (tested with Amber24), PDB2PQR (v3.6.1), and PDBTools (v2.5.1). Optional interaction analyses require PLIP v2.3.1. By default, the pipeline loads these dependencies as modulefiles. Users may alternatively modify the execution scripts to run with locally installed software, provided that all required executables are accessible through the system PATH.

**Molecular dynamics equilibration protocol**

All systems undergo a multi-stage relaxation and equilibration protocol prior to heated production simulations. Water molecules are first minimized for 1,000 steps using the steepest descent method, followed by 9,000 steps of conjugate gradient minimization under weak positional restraints of 1.0 kcal·mol⁻¹·Å⁻². Subsequently, a full system minimization is performed, consisting of 10,000 steps of steepest descent followed by 10,000 steps of conjugate gradient minimization without restraints. The system is then heated to 310 K under constant-volume (NVT) conditions for 0.5 ns, applying positional restraints of 100 kcal·mol⁻¹·Å⁻² to all atoms except hydrogens and water molecules. Water density equilibration is subsequently performed for 1.0 ns under constant pressure (1 bar) with identical restraints, after which restraints are reduced to 10 kcal·mol⁻¹·Å⁻² for an additional 1.0 ns equilibration stage. A new minimization of 10,000 conjugate gradient steps is then conducted with backbone-only positional restraints of 10 kcal·mol⁻¹·Å⁻². Gradual system release is achieved through three successive 1.0 ns NPT equilibration stages, during which backbone restraints are progressively reduced from 10 to 1 and finally to 0.1 kcal·mol⁻¹·Å⁻². The protocol concludes with 1.0 ns of fully unrestrained NPT molecular dynamics. Electrostatic, Lennard–Jones, and neighbor-list cutoffs are set to 8 Å throughout all equilibration stages. Production heated molecular dynamics (HMD) followed the protocol described by Radom *et al.* (2018), initiating at 310 K for 30 ns, followed by sequential temperature increases to 330 K and 360 K for 12.5 ns each, and finally to 390 K for the remaining 15 ns. All simulation steps were performed using the PMEMD engine of AMBER24, with temperature controlled via a Langevin thermostat and pressure regulated with a Monte Carlo barostat.

**Cross-validation and external validation of iRMSD descriptors**

Using the CAPRI quality tier assigned with DockQ v2, we assessed the ability of iRMSD-derived descriptors to discriminate between poses of different structural quality. The mean, maximum, and standard deviation of the iRMSD time series were calculated and averaged for each of the three HMD replicates. Comparison between CAPRI quality tiers was formulated as a binary classification problem: the higher-quality tier was defined as the positive class, including poses with descriptor values at or below the cutoff, reflecting greater preservation of the reference interface. Cutoff selection was performed independently for each iRMSD descriptor and CAPRI tier comparison. The robustness and generalizability of the resulting classification thresholds were evaluated using two group-based cross-validation schemes. In leave-one-complex-out (LOCO) cross-validation, each PDB complex was excluded in turn, with all poses from that complex reserved for evaluation. The cutoff was optimized using the remaining complexes and then applied to the excluded complex, ensuring that poses from the same complex were not used for both cutoff selection and evaluation. In leave-one-antigen-out (LOAO) cross-validation, the same procedure was performed at the antigen level, with all complexes associated with a given antigen excluded together. This provided a more stringent assessment of cutoff generalizability by evaluating its performance on antigens not represented during cutoff optimization. For each cross-validation scheme, predictions from all held-out folds were pooled to calculate accuracy and the Matthews correlation coefficient (MCC). Uncertainty in these estimates was quantified using 95% confidence intervals obtained from 2,000 bootstrap resamples performed at the corresponding grouping level: PDB complex for LOCO and antigen group for LOAO cross-validation. An additional external validation was performed to assess the transferability of the thresholds to complexes not involved in their development. The fixed cutoffs, established for each descriptor and CAPRI tier comparison, were applied without re-optimization to the additional complexes. Performance was evaluated both on the complete external validation set and on the External Novel subset, which contained only complexes involving antigens absent from the 30-complex development set.

**Supplementary Table 1.** DockQ scores for the benchmarking set of antibody-antigen complexes. For each PDB, four docking poses were selected based on their DockQ scores to represent high-quality (HQ), medium-quality (MQ), acceptable (ACC), and incorrect (INC) binding modes.

| **PDB ID** | **Antigen** | **DockQ** | | | |
| --- | --- | --- | --- | --- | --- |
|  |  | ***HQ*** | ***MQ*** | ***ACC*** | ***INC*** |
| 1A2Y | Lysozyme | 0.845 | 0.795 | 0.252 | 0.062 |
| 1MLC | Lysozyme | 0.863 | 0.505 | 0.255 | 0.042 |
| 1P2C | Lysozyme | 0.847 | 0.791 | 0.298 | 0.009 |
| 1PKQ | Myelin oligodendrocyte glycoprotein | 0.888 | 0.790 | 0.234 | 0.026 |
| 1R3I | Potassium channel KcsA | 0.861 | 0.744 | 0.244 | 0.091 |
| 1VFB | Lysozyme | 0.828 | 0.702 | 0.303 | 0.062 |
| 1WEJ | Cytochrome C | 0.866 | 0.716 | 0.260 | 0.081 |
| 1YQV | Lysozyme | 0.883 | 0.687 | 0.339 | 0.046 |
| 1ZTX | West Nile Virus E Protein | 0.860 | 0.771 | 0.230 | 0.052 |
| 2BOB | Potassium channel KcsA | 0.802 | 0.740 | 0.255 | 0.012 |
| 3AB0 | BclA major spore surface protein | 0.840 | 0.623 | 0.361 | 0.012 |
| 3FB6 | Potassium channel KcsA | 0.895 | 0.653 | 0.403 | 0.079 |
| 3HPL | Potassium channel KcsA | 0.881 | 0.598 | 0.282 | 0.048 |
| 3QWO | RSV fusion glycoprotein | 0.917 | 0.591 | 0.271 | 0.070 |
| 4F37 | Amyloid-β peptide | 0.806 | 0.732 | 0.365 | 0.018 |
| 4FFY | DenV E protein | 0.833 | 0.557 | 0.413 | 0.045 |
| 5CBA | CXCL13 | 0.878 | 0.792 | 0.470 | 0.055 |
| 5D8J | aP2 | 0.841 | 0.501 | 0.331 | 0.007 |
| 6A78 | Robo1 | 0.834 | 0.762 | 0.365 | 0.048 |
| 6FLA | DenV E protein | 0.820 | 0.583 | 0.273 | 0.082 |
| 7C01 | SARS-CoV-2 spike | 0.927 | 0.729 | 0.437 | 0.028 |
| 7DUO | CD38 | 0.841 | 0.750 | 0.332 | 0.022 |
| 7PHG | SARS-CoV-2 spike | 0.831 | 0.781 | 0.316 | 0.005 |
| 7QUH | Siglec-8 | 0.846 | 0.758 | 0.332 | 0.009 |
| 7Y1B | Basigin-2 | 0.921 | 0.567 | 0.302 | 0.042 |
| 8ATH | LAMP-1 | 0.895 | 0.589 | 0.287 | 0.007 |
| 8R9Z | PDCoV spike | 0.875 | 0.763 | 0.239 | 0.062 |
| 8TUI | KIR2DL3 | 0.913 | 0.515 | 0.281 | 0.075 |
| 9FKD | DBPro1156_2 binder | 0.818 | 0.782 | 0.443 | 0.065 |
| 9IMA | GPRC5D | 0.917 | 0.782 | 0.236 | 0.024 |

**Supplementary Table 2.** DockQ scores, pose classifications, and additional structural confidence scores for the Boltz-2 benchmarking set. PDB IDs and their respective antigens are listed alongside the DockQ scores used to categorize each structural pose, as well as ipSAE, pDockQ, pDockQ2, pTM, and ipTM scores.

| **PDB** | **Antigen** | **DockQ** | **Class** | **ipSAE** | **pDockQ** | **pDockQ2** | **pTM** | **ipTM** |
| --- | --- | --- | --- | --- | --- | --- | --- | --- |
| 1A2Y | Lysozyme | 0.013 | INC | 0.025 | 0.045 | 0.008 | 0.20 | 0.12 |
| 1KIP | Lysozyme | 0.022 | INC | 0.033 | 0.060 | 0.010 | 0.63 | 0.49 |
| 1ADQ | Autoantigen IgG Fc | 0.879 | HQ | 0.031 | 0.072 | 0.009 | 0.52 | 0.37 |
| 1ADQ | Autoantigen IgG Fc | 0.074 | INC | 0.030 | 0.046 | 0.009 | 0.51 | 0.36 |
| 2BOB | Potassium channel KcsA | 0.976 | HQ | 0.032 | 0.047 | 0.009 | 0.32 | 0.21 |
| 2QQN | Neuropilin-1 | 0.912 | HQ | 0.029 | 0.040 | 0.008 | 0.22 | 0.10 |
| 6FLA | DenV E protein | 0.936 | HQ | 0.023 | 0.048 | 0.008 | 0.32 | 0.22 |
| 6PLK | ZIKV envelope DIII | 0.852 | HQ | 0.035 | 0.095 | 0.013 | 0.68 | 0.54 |
| 6YE3 | IL-2 | 0.921 | HQ | 0.755 | 0.218 | 0.848 | 0.92 | 0.90 |
| 6L62 | Porcine Circovirus type 2 Cap protein | 0.011 | INC | 0.023 | 0.065 | 0.010 | 0.56 | 0.41 |
| 9FKD | DBPro1156_2 binder | 0.399 | ACC | 0.024 | 0.047 | 0.008 | 0.22 | 0.15 |
| 9FKD | DBPro1156_2 binder | 0.040 | INC | 0.025 | 0.044 | 0.008 | 0.22 | 0.14 |

**Supplementary Table 3.** Composition of external redocking validation set. Complexes not included in the 30-complex benchmark, used to validate the locked thresholds. For each complex, the antigen was qualified into novel if absent from the initial benchmark set.

| **PDB** | **Antigen** | **Novel antigen** | **Poses** | **Pose 1 DockQ** | **Pose 1 iRMSD** | **Pose 2 DockQ** | **Pose 2 iRMSD** | **Pose 3 DockQ** | **Pose 3 iRMSD** |
| --- | --- | --- | --- | --- | --- | --- | --- | --- | --- |
| 5GGV | CTLA-4 | Yes | 1 | 0.427 | 2.984 | - | - | - | - |
| 1YY9 | EGFR | Yes | 1 | 0.010 | 16.286 | - | - | - | - |
| 5T5F | Factor H | Yes | 2 | 0.887 | 0.772 | 0.106 | 7.318 | - | - |
| 5OB5 | GroBeta | Yes | 2 | 0.860 | 0.846 | 0.082 | 6.201 | - | - |
| 3P11 | HER3 | Yes | 1 | 0.873 | 0.768 | - | - | - | - |
| 4G6J | IL-1β | Yes | 3 | 0.810 | 0.997 | 0.323 | 2.864 | 0.032 | 7.835 |
| 3G6D | IL-13 | Yes | 1 | 0.077 | 9.720 | - | - | - | - |
| 4YPG | Interferon | Yes | 3 | 0.882 | 0.783 | 0.358 | 3.208 | 0.022 | 14.300 |
| 6IAP | NKP46 | Yes | 2 | 0.832 | 0.936 | 0.057 | 9.003 | - | - |
| 5LQB | Proleukin | Yes | 1 | 0.009 | 13.524 | - | - | - | - |
| 3MXW | Sonic hedgehog | Yes | 1 | 0.879 | 0.903 | - | - | - | - |
| 5VQM | TcdB-GTD | Yes | 2 | 0.889 | 0.754 | 0.023 | 10.612 | - | - |
| 6J5F | TBE E protein | Yes | 2 | 0.889 | 0.604 | 0.056 | 10.670 | - | - |
| 1K4D | Potassium channel KcsA | No | 1 | 0.037 | 8.595 | - | - | - | - |
| 2DWE | Potassium channel KcsA | No | 1 | 0.029 | 10.315 | - | - | - | - |

**Supplementary Table 4.** Analysis of high-quality (HQ), medium-quality (MQ), acceptable (ACC), and incorrect (INC) docking poses with energy-based (HADDOCK score and REF15) and AI-based (DeepRank-GNN-ESM and GNN-Dove) scoring functions.

| **PDB** | **HADDOCK Score** | | | | | **REF15** | | | **DeepRank-GNN-ESM** | | | | | | **GNN-Dove** | | | | |  |
| --- | --- | --- | --- | --- | --- | --- | --- | --- | --- | --- | --- | --- | --- | --- | --- | --- | --- | --- | --- | --- |
|  | **HQ** | **MQ** | **ACC** | **INC** | **HQ** | | **MQ** | **ACC** | | **INC** | **HQ** | **MQ** | **ACC** | **INC** | | **HQ** | **MQ** | **ACC** | **INC** | |
| 1A2Y | -245.7 | -220.2 | -241.0 | -240.2 | 77.2 | | 294.6 | 179.8 | | 299.2 | 0.02 | 0.10 | 0.07 | 0.04 | | 0.10 | 0.08 | 0.17 | 0.09 | |
| 1MLC | -238.1 | -214.4 | -200.7 | -179.0 | 415.6 | | 673.6 | 492.2 | | 483.2 | 0.14 | 0.06 | 0.07 | 0.04 | | 0.23 | 0.23 | 0.23 | 0.23 | |
| 1P2C | -249.7 | -254.5 | -217.9 | -249.8 | 292.0 | | 698.9 | 696.5 | | 743.0 | 0.11 | 0.11 | 0.11 | 0.12 | | 0.23 | 0.23 | 0.23 | 0.13 | |
| 1PKQ | -252.7 | -242.0 | -146.7 | -155.5 | 705.7 | | 725.5 | 719.4 | | 627.5 | 0.40 | 0.49 | 0.19 | 0.06 | | 0.09 | 0.11 | 0.12 | 0.16 | |
| 1R3I | -299.9 | -279.2 | -228.2 | -233.7 | 216.4 | | 183.0 | 347.1 | | 249.9 | 0.27 | 0.22 | 0.04 | 0.23 | | 0.10 | 0.11 | 0.23 | 0.23 | |
| 1VFB | -250.5 | -204.8 | -217.5 | -243.6 | 234.7 | | 465.2 | 273.7 | | 329.6 | 0.08 | 0.07 | 0.02 | 0.07 | | 0.10 | 0.16 | 0.18 | 0.07 | |
| 1WEJ | -275.0 | -278.5 | -252.7 | -241.6 | 474.1 | | 577.4 | 349.7 | | 584.7 | 0.02 | 0.03 | 0.01 | 0.15 | | 0.11 | 0.20 | 0.18 | 0.21 | |
| 1YQV | -250.2 | -245.9 | -201.5 | -210.2 | 428.4 | | 554.1 | 427.5 | | 35.2 | 0.23 | 0.19 | 0.16 | 0.20 | | 0.15 | 0.26 | 0.23 | 0.23 | |
| 1ZTX | -265.0 | -269.0 | -205.7 | -209.7 | 483.5 | | 466.2 | 454.7 | | 704.0 | 0.15 | 0.10 | 0.07 | 0.19 | | 0.14 | 0.12 | 0.12 | 0.23 | |
| 2BOB | -279.9 | -281.8 | -215.7 | -214.9 | 452.3 | | 216.5 | 457.7 | | 504.0 | 0.27 | 0.21 | 0.07 | 0.04 | | 0.09 | 0.09 | 0.27 | 0.09 | |
| 3AB0 | -288.7 | -265.1 | -233.0 | -191.9 | 589.9 | | 413.4 | 569.0 | | 418.8 | 0.13 | 0.11 | 0.03 | 0.13 | | 0.24 | 0.17 | 0.14 | 0.23 | |
| 3FB6 | -291.5 | -272.8 | -249.8 | -230.1 | 115.2 | | 156.0 | 172.7 | | 531.3 | 0.35 | 0.22 | 0.14 | 0.47 | | 0.08 | 0.09 | 0.13 | 0.11 | |
| 3HPL | -289.7 | -265.2 | -246.2 | -207.5 | 395.5 | | 347.4 | 446.4 | | 372.8 | 0.29 | 0.22 | 0.27 | 0.38 | | 0.08 | 0.10 | 0.08 | 0.11 | |
| 3QWO | -267.6 | -255.8 | -228.4 | -246.3 | -127.9 | | -14.1 | 192.7 | | 208.5 | 0.14 | 0.10 | 0.05 | 0.03 | | 0.12 | 0.16 | 0.22 | 0.26 | |
| 4F37 | -257.4 | -255.4 | -238.3 | -240.0 | 1008.9 | | 987.6 | 826.9 | | 957.2 | 0.47 | 0.42 | 0.47 | 0.22 | | 0.08 | 0.18 | 0.09 | 0.20 | |
| 4FFY | -303.3 | -289.0 | -290.5 | -231.2 | 241.5 | | 241.5 | 209.8 | | 223.7 | 0.27 | 0.33 | 0.29 | 0.31 | | 0.10 | 0.13 | 0.11 | 0.23 | |
| 5CBA | -293.4 | -309.6 | -270.8 | -221.1 | 333.8 | | 383.4 | 453.2 | | 409.2 | 0.68 | 0.37 | 0.21 | 0.49 | | 0.06 | 0.06 | 0.07 | 0.08 | |
| 5D8J | -319.4 | -246.2 | -284.2 | -228.7 | 880.2 | | 855.1 | 908.4 | | 525.4 | 0.16 | 0.14 | 0.23 | 0.03 | | 0.09 | 0.23 | 0.10 | 0.14 | |
| 6A78 | -258.8 | -235.5 | -232.1 | -207.2 | 278.8 | | 260.0 | 363.8 | | 303.2 | 0.27 | 0.26 | 0.25 | 0.17 | | 0.19 | 0.17 | 0.21 | 0.12 | |
| 6FLA | -274.3 | -252.0 | -222.6 | -198.2 | 607.9 | | 717.2 | 444.1 | | 568.3 | 0.20 | 0.11 | 0.07 | 0.16 | | 0.19 | 0.13 | 0.22 | 0.18 | |
| 7C01 | -325.8 | -321.0 | -272.1 | -240.2 | 421.8 | | 461.4 | 634.2 | | 518.9 | 0.32 | 0.33 | 0.40 | 0.40 | | 0.23 | 0.23 | 0.23 | 0.10 | |
| 7DUO | -261.7 | -248.4 | -213.4 | -231.6 | 700.4 | | 739.8 | 648.6 | | 276.5 | 0.19 | 0.21 | 0.14 | 0.22 | | 0.13 | 0.13 | 0.13 | 0.18 | |
| 7PHG | -248.2 | -249.9 | -223.0 | -201.7 | 1899.6 | | 1842.6 | 2042.9 | | 1873.2 | 0.72 | 0.77 | 0.56 | 0.30 | | 0.23 | 0.23 | 0.23 | 0.21 | |
| 7QUH | -246.8 | -248.2 | -233.1 | -229.6 | 261.1 | | 527.6 | 198.6 | | 237.5 | 0.33 | 0.35 | 0.34 | 0.29 | | 0.09 | 0.11 | 0.26 | 0.12 | |
| 7Y1B | -277.2 | -221.9 | -223.5 | -198.1 | 680.2 | | 836.3 | 796.9 | | 735.1 | 0.12 | 0.04 | 0.09 | 0.10 | | 0.29 | 0.31 | 0.28 | 0.26 | |
| 8ATH | -293.7 | -272.1 | -272.3 | -245.4 | 674.4 | | 585.6 | 710.5 | | 400.5 | 0.32 | 0.36 | 0.25 | 0.36 | | 0.18 | 0.17 | 0.16 | 0.20 | |
| 8R9Z | -197.5 | -219.1 | -191.1 | -198.4 | 506.1 | | 473.9 | 725.4 | | 633.9 | 0.19 | 0.27 | 0.32 | 0.07 | | 0.23 | 0.23 | 0.23 | 0.23 | |
| 8TUI | -340.2 | -304.8 | -262.5 | -265.6 | 384.4 | | 352.9 | 218.8 | | 306.3 | 0.65 | 0.46 | 0.54 | 0.33 | | 0.12 | 0.15 | 0.14 | 0.23 | |
| 9FKD | -235.7 | -246.8 | -234.7 | -184.0 | 185.2 | | 346.8 | 421.2 | | 105.2 | 0.29 | 0.39 | 0.28 | 0.05 | | 0.20 | 0.21 | 0.17 | 0.23 | |
| 9IMA | -247.7 | -244.8 | -247.9 | -231.6 | 435.0 | | 308.3 | 133.4 | | 445.5 | 0.29 | 0.30 | 0.19 | 0.60 | | 0.23 | 0.23 | 0.23 | 0.23 | |

**Supplementary Table 5.** Cross-validated and external classification of iRMSD descriptors across CAPRI quality tiers. In each comparison, the higher-quality tier is the positive class, and a pose is assigned to it when its descriptor value is at or below the reshold. Accuracy (Acc, %) and Matthews correlation coefficient (MCC) under leave-one-complex-out (LOCO) and leave-one-antigen-out (LOAO) cross-validation, and on an external redocking set. Values in brackets correspond to the 95% confidence interval of the MCC, estimated from 2,000 bootstrap resamples over the grouping unit. The external set was further refined into a subset (External Novel) comprising antigens not represented in the initial evaluation.

| **Comparison** | **Descriptor** | **Threshold (Å)** | **LOCO Acc / MCC [95% CI]** | **LOAO Acc / MCC [95% CI]** | **External All Acc / MCC (n)** | **External Novel Acc / MCC (n)** |
| --- | --- | --- | --- | --- | --- | --- |
| HQ vs. INC | Mean iRMSD | 2.52 ± 0.02 | 90.0 / 0.800  [0.667-0.935] | 90.0 / 0.800  [0.592-0.946] | 95.2 / 0.908 (21) | 94.7 / 0.900 |
| HQ vs. INC | Maximun iRMSD | 5.98 ± 0.27 | 83.3 / 0.667  [0.500-0.834] | 81.7 / 0.634  [0.467-0.789] | 85.7 / 0.750 (21) | 89.5 / 0.809 |
| HQ vs. INC | Std. dev. iRMSD | 0.96 ± 0.07 | 83.3 / 0.667  [0.500-0.834] | 81.7 / 0.634  [0.465-0.807] | 85.7 / 0.750 (21) | 89.5 / 0.809 |
| HQ vs. ACC | Mean iRMSD | 2.40 ± 0.08 | 78.3 / 0.567  [0.369–0.767] | 78.3 / 0.567  [0.325–0.782] | 100 / 1.000  (12) | 100 / 1.000  (12) |
| HQ vs. ACC | Maximun iRMSD | 3.64 ± 0.03 | 80.0 / 0.636  [0.452–0.816] | 80.0 / 0.636  [0.410–0.811] | 66.7 / 0.488 (12) | 66.7 / 0.488  (12) |
| HQ vs. ACC | Std. dev. iRMSD | 0.48 ± 0.04 | 71.7 / 0.446  [0.244–0.642] | 73.3 / 0.476  [0.277–0.642] | 66.7 / 0.488 (12) | 66.7 / 0.488  (12) |
| HQ vs. MQ | Mean iRMSD | 1.59 ± 0.02 | 61.7 / 0.251  [0.069-0.446] | 61.7 / 0.251  [0.100–0.422] | - | - |
| HQ vs. MQ | Maximun iRMSD | 2.41 ± 0.00 | 58.3 / 0.260  [0.047–0.447] | 51.7 / 0.043 [−0.140–0.264] | - | - |
| HQ vs. MQ | Std. dev. iRMSD | 0.47 ± 0.00 | 56.7 / 0.138  [−0.037–0.336] | 56.7 / 0.138 [−0.040–0.353] | - | - |
| ACC vs. INC | Mean iRMSD | 4.09 ± 1.96 | 46.7 / −0.067 [−0.233–0.100] | 46.7 / −0.068 [−0.240–0.089] | 80.0 / 0.612 (15) | 84.6 / 0.693  (13) |
| ACC vs. INC | Maximun iRMSD | 7.69 ± 0.96 | 60.0 / 0.200  [0.000–0.434] | 60.0 / 0.200  [0.000–0.409] | 80.0 / 0.612 (15) | 84.6 / 0.693  (13) |
| ACC vs. INC | Std. dev. iRMSD | 1.14 ± 0.00 | 73.3 / 0.476  [0.277–0.680] | 73.3 / 0.476  [0.282–0.696] | 73.3 / 0.354 (15) | 76.9 / 0.426  (13) |

**Supplementary Figure 1.** Antibody sequence clustering on the docking benchmark dataset. Each box represents a distinct cluster, with orange nodes identifying the representative sequence, and blue nodes indicating additional members sharing high sequence identity.

**Supplementary Figure 2.** Docking pose quality across representative antibody-antigen complexes. Structural overlays of docking predictions against the crystal structure (grey) for (A) Lysozyme-D1.3 complex (1A2Y), (B) CXCL13-3B4 complex (5CBA), and (C) LAMP1-FAB-B complex (8ATH). Poses are categorized by DockQ scores into high-quality (green), medium-quality (blue), acceptable (yellow), and incorrect (red) binding modes. The antigen is shown in blue.

**Supplementary Figure 3.** Computational performance of the HMD protocol. Runtime in hours is plotted against system size (number of residues) for various NVIDIA GPU architectures. Individual data points represent independent benchmarking simulations, color-coded by GPU device. The dashed red line illustrates the linear scaling of runtime relative to system size.

**Supplementary Figure 4.** iRMSD distributions across CAPRI-based pose quality categories. The iRMSD values relative to the corresponding crystal structures for high-quality (HQ), medium (MQ), acceptable (ACC), and incorrect (INC) poses were extracted using DockQ v2. Lower iRMSD values indicate greater interface similarity to the reference crystal structures.

**Supplementary Figure 5.** Impact of temperature stepping on antibody-antigen interface stability. Mean iRMSD values for high-quality (green), medium (blue), acceptable (yellow), and incorrect (red) docking poses across four successive temperature windows (310, 330, 360, and 390 K). Error bars represent standard deviation.

**Supplementary Figure 6.** Spearman correlation analysis between DockQ scores and AI- or energy-based scoring metrics across the benchmarking set. Correlations are shown for HADDOCK score, REF15, DeepRank-GNN-ESM, GNN-DOVE, mean iRMSD, and mean iRMSD at 390 K. High-quality (green), medium (blue), acceptable (yellow), and incorrect (red) poses are indicated according to their CAPRI-based classification.

**Supplementary Figure 7.** HMD simulation stability of native crystal structures. Interface RMSD (iRMSD) profiles across three independent replicas for (A) the P5C3-RBD complex (PDB ID: 7PHG) and (B) the anti-peptide A antibody complex (PDB ID: 4F37). The dashed horizontal line at 5 Å serves as a reference for structural stability.

**Supplementary Figure 8.** Structural and interaction analysis of antibody–antigen interfaces before and after alanine mutations. (A) Interface of the high-quality pose for PDB 7C01 (CB6–RBD), with the antibody heavy and light chains shown in dark and light blue, respectively, and the RBD in gray. Key interface residues selected for alanine substitution are highlighted, with iRMSD values for the wild-type (WT) and mutated complexes shown below. (B) PLIP interaction map of the high-quality pose before mutation and (C) after substitution of Asp100 and Tyr92 with alanine. (D) Incorrect pose of 7C01, with the heavy and light chains shown in red and salmon, respectively, and corresponding iRMSD values after mutation. (E) PLIP interaction map of the incorrect pose before mutation and (F) after mutation of the same residues. (G) High-quality pose of PDB 9IMA (GPRC5D–talquetamab), with the antibody heavy and light chains shown in dark and light blue, respectively, and the antigen in gray. (H) PLIP interaction map before mutation and (I) after substitution of Arg98 with alanine. (J) Incorrect pose of 9IMA, with the antibody heavy and light chains shown in red and salmon, respectively, and corresponding iRMSD values before and after mutation of Arg98 to alanine. (K) PLIP interaction map of the incorrect pose before mutation and (L) after substitution of Arg98 with alanine. In interaction maps, orange, blue, red, purple, and green lines indicate hydrophobic interactions, hydrogen bonds, salt bridges, π–π stacking, and π-cation interactions, respectively; line thickness represents interaction prevalence.

**Supplementary Figure 9.** Descriptive statistics of iRMSD distributions for docking pose classification. Violin plots with overlaid boxplots illustrate the distribution of (A) mean iRMSD, (B) maximum iRMSD, and (C) iRMSD standard deviation across the benchmarking set. High-quality (green), medium (blue), acceptable (yellow), and incorrect (red) pose groups were assessed. (D) Classification performance for high-quality versus incorrect poses as a function of the number of HMD replicates (1–3), using the previously established 5 Å maximum iRMSD cutoff. Points and error bars represent the mean and standard deviation of accuracy (left axis) and MCC (right axis) across replicate combinations.

**Supplementary Figure 10.** Dynamic interaction analysis of docking poses during HMD simulations. Violin plots and individual data points characterize the binding interface behavior for high-quality (green), medium (blue), acceptable (yellow), and incorrect (red) poses across four metrics: (A) total number of interactions, (B) number of unique residues involved in the interface, (C) mean interaction duration as a percentage of total simulation time, and (D) number of persistent interactions (present in >50% of the simulation). Asterisks indicate levels of statistical significance between groups (*p < 0.05, **p < 0.01, ***p < 0.001).

**Supplementary Figure 11.** Dynamic interface stability through unique interaction tracking. Time-course analysis of the number of unique molecular interactions for representative antibody-antigen complexes across high-quality (green), acceptable (yellow), and incorrect (red) docking poses. Each subplot displays 525 total frames, representing three independent MD replicates (175 frames per replicate) concatenated sequentially. The black dashed line indicates the rolling average (window = 26), while the red dotted line represents the global mean for the entire simulation. (A–R) Results for complexes 1YQV, 3HPL, 4F37, 7C01, 8ATH, and 8R9Z.

**Supplementary Figure 12.** ROC curve analysis of iRMSD metrics for docking pose classification. Receiver Operating Characteristic (ROC) curves evaluate the discriminative power of mean (green), maximum (purple), and standard deviation (orange) iRMSD values. Classifications are shown for (A) high-quality versus incorrect poses, (B) high-quality versus medium-quality poses, (C) high-quality versus acceptable poses, and (D) acceptable versus incorrect poses. Area Under the Curve (AUC) values are provided in the legends, with the dashed diagonal line representing random performance (AUC = 0.500).

**Supplementary Figure 13.** Validation of AutoHMD on Boltz-2-derived poses. Heatmaps displaying interfacial RMSD (iRMSD) over time (0–70 ns) for poses categorized as high-quality (top), acceptable (middle), and incorrect (bottom). Each heatmap shows the evolution of five independent replicates across the four temperature windows, with the color scale (0 to 12 Å) indicating structural deviation from the initial modeled conformation.

**Supplementary Figure 14**. Distribution of iRMSD descriptive statistics for the Boltz-2 set, showing (A) mean iRMSD, (B) iRMSD standard deviation, and (C) maximum iRMSD across high-quality, acceptable, and incorrect poses. (D) Comparison of mean iRMSD values across the four temperature windows. Error bars represent the standard deviation among trajectories.

**Supplementary Figure 15**. Transferability of benchmark-derived iRMSD cutoffs to Boltz-2 predicted models. Maximum (A), mean (B), and iRMSD standard deviation (C) descriptors, averaged over five HMD replicates, are shown for 15 Boltz-2 poses (7 high-quality and 8 incorrect). Dashed lines indicate the benchmark-derived cutoffs (5.98, 2.52, and 0.96 Å, respectively), with open circles marking misclassified poses. (D, E) Impact of replicate number on classification performance using benchmark-derived iRMSD cutoffs. Per-pose mean, maximum, and standard-deviation iRMSD values were averaged over increasing numbers of HMD replicates (k = 1–5) and classified using fixed benchmark-derived cutoffs. Points and error bars represent the mean ± SD of (D) accuracy and (E) MCC.

**Supplementary Figure 16.** Interface interaction profiling for Boltz-2 poses during HMD simulations. Distribution of interface metrics for high-quality (green), acceptable (yellow), and incorrect (red) poses derived from the Boltz-2 dataset. Metrics include (A) total number of interactions, (B) number of unique interfacial residues, (C) mean interaction duration across the simulation, and (D) number of persistent interactions (present in >50% of frames). Statistical significance between the high-quality and incorrect groups is indicated in panel D (**p < 0.01).
